# Influence of tobacco smoking and alcohol drinking on anxiety in sample of 30 836 individuals in Taiwan Biobank

**DOI:** 10.1192/bjo.2025.24

**Published:** 2025-04-01

**Authors:** Ya-Ting Wen, Kuan-Po Lien, Jerry Cheng-Yen Lai, Shaw-Ji Chen

**Affiliations:** Department of Medicine, Mackay Medical College, New Taipei City, Taiwan; Department of Dermatology, Taichung Veterans General Hospital, Taichung, Taiwan; Department of Surgery, Cathay General Hospital, Taipei, Taiwan; Department of Medical Research, Taitung MacKay Memorial Hospital, Taitung, Taiwan; Master Programme in Biomedicine, National Taitung University, Taitung, Taiwan; Department of Psychiatry, Taitung MacKay Memorial Hospital, Taitung, Taiwan

**Keywords:** Taiwan Biobank, anxiety, alcohol, tobacco, sex

## Abstract

**Background:**

Anxiety disorders are among the most common mental disorders worldwide, and most previous studies have focused solely on alcohol drinking or tobacco smoking as risk factors for anxiety.

**Aim:**

This study investigated the associations of alcohol drinking and tobacco smoking with anxiety.

**Method:**

The data of 30 836 individuals in the Taiwan Biobank were retrieved and analysed in our study. To investigate the associations of tobacco and alcohol use with anxiety, we analysed Patient Health Questionnaire 4 (specifically scores for the first two questions assessing generalised anxiety disorder) results of the included participants and data on their tobacco and alcohol use, and other covariates.

**Results:**

Participants who used only tobacco and those using both tobacco and alcohol were more likely to experience anxiety than were those who did not use tobacco or alcohol. Among men, the use of alcohol and/or tobacco was associated with a significantly higher risk of anxiety. Among women, the use of both alcohol and tobacco was associated with a significantly higher risk of anxiety. Older age was associated with a lower risk of anxiety.

**Conclusions:**

Tobacco and alcohol use significantly influence the risk of anxiety, particularly in men, and older age also influences this risk. The associations of anxiety with tobacco and alcohol use in women may change because of the increasing prevalence of their use among women in Taiwan in recent years.

## Introduction

Anxiety disorders are among the most common types of mental disorder worldwide. The National Comorbidity Survey Replication reported that, from 2001 to 2003, an estimated 19.1% of the adult population in the USA had anxiety disorder, and it estimated that the lifetime morbid risk of the general population was 31.1%.^1^ The prevalence of common mental disorders, including anxiety disorders, also increased from 11.5% in 1990 to 23.8% in 2010 in Taiwan.^2^ The risk factors for anxiety disorder have been extensively explored and discussed, and alcohol use and/or tobacco use have been examined as risk factors for anxiety in previous studies.^3,4^ Although some studies have proposed several candidate mechanisms underlying how alcohol or tobacco increases anxiety, further clarification is required.^5,6^ Another key topic is whether alcohol use and tobacco use are caused by anxiety or vice versa. Because alcohol and tobacco are regularly consumed,^7,8^ anxiety interventions that target these substances have considerable utility to public health.

In addition to alcohol and tobacco use, other factors may affect anxiety (e.g. sex, age, exercise habits, coffee or tea drinking, sleep profile and family history of mental illness). The prevalence of anxiety disorder has been reported to be higher among women (women versus men, 23.4 and 14.3%, respectively).^1,4^ Some studies have indicated that younger age is associated with a higher risk of anxiety disorders.^9^ Regular exercise was identified as a protective factor against anxiety disorders.^10^ Although caffeine consumption has long been proposed and studied as either a protective or risk factor for various psychiatric disorders, most studies have reported that caffeine intake exacerbates the symptoms of anxiety and agitation.^11,12^ Tea, which contains L-theanine (an amino acid that can enhance cognitive function), is widely consumed, and studies have suggested that tea consumption reduces the symptoms of anxiety.^12,13^ Furthermore, adequate sleep is crucial to various aspects of human health.^14,15^ Studies have suggested that sleep deprivation increases the risk of mental illnesses such as anxiety.^14^ In addition, a family history of mental illness should be considered during the evaluation of anxiety risk.^16,17^ The identification of these associations can help people modify their lifestyle habits and safeguard their mental health.

This study investigated the association of alcohol and tobacco use with anxiety. We also identified various risk and protective factors for anxiety in people from different sex and age groups who either use tobacco and alcohol or not.

## Method

### Taiwan Biobank

The Taiwan Biobank (TWB) was established by Taiwan’s government to collect lifestyle and genetic data on the Taiwanese population.^18–20^ Those in the TWB programme volunteer to participate, and they comprise individuals aged between 30 and 70 years without a history of cancer. After signing an informed consent form, participants in the TWB programme provide information regarding their daily lives, blood samples and physical examination results. In addition, each participant completes a questionnaire administered during a face-to-face interview with a TWB researcher. The questionnaire pertains to the participants’ personal information and lifestyles.

### Study instruments

#### Anxiety measurement tool

In the TWB, Patient Health Questionnaire 4 (PHQ-4) was used to assess participants’ levels of depression and anxiety. In the present study, participants were defined as having anxiety disorder if they had a total score of ≥3 for the first two questions of PHQ-4, based on a two-item anxiety scale (Generalized Anxiety Disorder 2-item).^21^

#### Alcohol and tobacco use

The TWB participants included in the present study were classified as alcohol users if they reported a weekly alcohol intake of >150 mL for at least 6 months and were still using alcohol when they completed PHQ-4. Those who had smoked regularly for at least 6 months and were still smoking when they completed PHQ-4 were classified as tobacco users.

### Covariates

This study consulted the literature and included controls for sex, age, body mass index (BMI; weight (kg)/height (m)), exercise habits, educational attainment, sleep profile (including total sleep time on weekdays and weekends), social jet lag (SJL) between weekdays and weekends, sleep quality and family history of mental illness. Among the sleep quality data retrieved from the TWB, sleep quality was originally rated as either very good, good, average, bad or very bad. However, in our analysis, sleep quality was reclassified as either good^+^ (very good, good and average) or bad^+^ (bad and very bad). The concept of SJL refers to differences in sleep patterns between weekdays (workdays) and weekends (sleep hour difference between weekdays and weekends). SJL was revealed to be related to psychiatric disorders, including anxiety.^22^ Furthermore, we collected data on whether menopause had occurred in women participating in the TWB.

The demographic data comprised sex, age, exercise habits, educational attainment and sleep profile; these data were obtained from participants via interviews conducted through the TWB programme. The TWB contains information on body height and weight of each participant, enabling calculation of their BMI.^2^

### Ethics statement

Establishment of the TWB was approved by the Institutional Review Board of Biomedical Science Research/IRB-BM, Academia Sinica, Taiwan, and by the Ethics and Governance Council of Taiwan Biobank, Taiwan. Written informed consent was obtained from all TWB participants in accordance with official regulations and the principles of the Declaration of Helsinki. The present study was also approved by the Institutional Review Board of MacKay Memorial Hospital (IRB no. 21MMHIS351e).

### Statistical analysis

Participant characteristics were described using means (standard deviation, s.d.) for continuous variables and frequencies (percentages) for categorical variables; odds ratios with 95% CIs were used to describe differences between the use versus non-use of alcohol and/or tobacco. All variables were compared using one-way analysis of variance for continuous data, and *χ*^2^ tests (or Fisher’s exact test) for categorical data. Participant characteristics with a *P*-value of <0.05 were included in the logistic regression model, which was used to determine the covariate-adjusted odds ratio for the risk of anxiety. All data transformations and statistical analyses were conducted using the statistical software SAS for Windows (version 9.4; SAS Institute, Cary, NC, USA). A null hypothesis was rejected at an alpha level of 0.05.

## Results

### Basic characteristics of TWB participants

Following the exclusion of respondents aged <40 years (*n* = 3201) and those with incomplete or missing information related to independent variables of interest (*n* = 1093), 27 916 participants were included in our analysis. All data pertaining to the included participants (i.e. PHQ-4 results, alcohol and tobacco use, sex, age, BMI, exercise habits, coffee and tea consumption, education level, residential urbanicity, family history and sleeping profile) are listed in Table 1.

**Table 1:** Baseline characteristics of all follow-up participants stratified by alcohol drinking and smoking behaviors (*N* = 30 836)

	Non-users of alcohol and tobacco	Tobacco-only users	Alcohol-only users	Users of alcohol and tobacco	*P*-value
Baseline characteristics	(*n* = 21 924, 71.1%)	(*n* = 5564, 18.0%)	(*n* = 961, 3.1%)	(*n* = 2387, 7.7%)	
Independent variables					
Male (%)	4124 (18.8)	4309 (77.4)	526 (54.7)	2183 (91.5)	<0.001***
Age (years), mean (s.d.)	55.3 (10.3)	54.6 (10.6)	55.1 (10.8)	55.6 (9.8)	<0.001***
<55, *n* (%)	9294 (42.4)	2604 (46.8)	416 (43.3)	996 (41.7)	<0.001***
≥55	12 630 (57.6)	2960 (53.2)	545 (56.7)	1391 (58.3)	
Residential urbanicity, *n* (%)					
Rural	1247 (5.7)	350 (6.3)	55 (5.7)	206 (8.6)	<0.001
Non-rural	20 677 (94.3)	5214 (93.7)	906 (94.3)	2181 (91.4)	
Education level, *n* (%)					
College or graduate school	11 040 (50.4)	3047 (54.8)	490 (51.0)	1140 (47.8)	<0.001***
High school, elementary school or lower	10 884 (49.6)	2517 (45.2)	471 (49.0)	1247 (52.2)	
BMI, mean (s.d.)	24.0 (3.7)	25.2 (3.6)	24.7 (3.5)	25.6 (3.5)	<0.001***
BMI, *n* (%)					
<25	14 472 (66.0)	2865 (51.5)	552 (57.4)	1083 (45.4)	<0.001
≥25	7452 (34.0)	2699 (48.5)	409 (42.6)	1304 (54.6)	
Behavioural factors, *n* (%)					
Regular exercise	11 307 (51.6)	3097 (55.7)	471 (49.0)	1254 (52.5)	<0.001
Coffee consumption	8947 (40.8)	2655 (47.7)	499 (51.9)	1104 (46.3)	<0.001***
Tea consumption	4127 (18.8)	1761 (31.6)	308 (32.0)	916 (38.4)	<0.001***
Sleep profile					
Sleep hours (weekdays)					
Total hours, mean (s.d.)	6.60 (1.09)	6.65 (1.09)	6.68 (1.17)	6.65 (1.18)	<0.001***
Sleep time (24 h), *n* (%)					
Before midnight	17 139 (78.2)	3992 (71.7)	701 (72.9)	1663 (69.7)	<0.001
After midnight	4785 (21.8)	1572 (28.3)	260 (27.1)	724 (30.3)	
Sleep hours (weekends), *n* (%)					
Total hours, mean (s.d.)	6.98 (1.32)	7.10 (1.37)	7.02 (1.42)	7.04 (1.42)	<0.001***
Sleep time (24 h)					
Before midnight	16 669 (76.0)	3852 (69.2)	674 (70.1)	1615 (67.7)	<0.001
After midnight	5255 (24.0)	1712 (30.8)	287 (29.9)	772 (32.3)	
Social jet lag between weekdays and weekends					
Difference in sleep hours, mean (s.d.)	0.42 (0.88)	0.51 (1.01)	0.37 (0.99)	0.43 (0.99)	<0.001***
Difference in sleep hours >1 h, *n* (%)	5155 (23.5)	1441 (25.9)	186 (19.4)	515 (21.6)	<0.001***
Sleep quality, *n* (%)					
Very bad + bad (bad^+^)	4485 (20.5)	1046 (18.8)	222 (23.1)	481 (20.2)	0.005**
Average + good + very good (good^+^)	17 439 (79.5)	4518 (81.2)	739 (76.9)	1906 (79.8)	
Family history of mental illness	862 (3.9)	146 (2.6)	37 (3.9)	66 (2.8)	<0.001***
PHQ-4 score, mean (s.d.)	0.76 (1.47)	0.74 (1.52)	0.73 (1.54)	0.75 (1.56)	0.743
Presence of anxiety based on PHQ-4, *n* (%)	794 (3.6)	185 (3.3)	42 (4.4)	90 (3.8)	0.379

s.d., standard deviation.

*P < 0.05, **P < 0.01, ***P < 0.001 (*P*-value, *χ*^2^ test or one-way analysis of variance test).

The included participants were classified into four groups based on smoking and drinking status: non-users of both alcohol and tobacco (*n* = 19 889, 71.2%), tobacco-only users (*n* = 4947, 17.7%), alcohol-only users (*n* = 858, 3.1%) and users of both alcohol and tobacco (*n* = 2222, 8.0%). Mean (s.d.) PHQ-4 scores for the four groups were as follows: non-users of both alcohol and tobacco, 0.76 (1.47); tobacco-only users, 0.74 (1.52); alcohol-only users, 0.73 (1.54); and users of both alcohol and tobacco, 0.75 (1.56). Because PHQ-4 scores in the TWB were low in our survey, we added binary measures of anxiety. In accordance with the previous study,^21^ a participant was defined as having anxiety disorder if they had a total score of ≥3 for the first two questions in PHQ-4. The prevalence of anxiety according to PHQ-4 scores of the four groups was as follows: non-users of both alcohol and tobacco, 794 (3.6%); tobacco-only users, 185 (3.3%); alcohol-only users, 42 (4.4%); and users of both alcohol and tobacco, 90 (3.8%). That is, anxiety based on PHQ-4 scores did not differ significantly between alcohol and tobacco users in the univariate analysis. Therefore, we used logistic regression for a multivariate test, as recommended in previous studies.^23,24^

Significant covariates with *P* < 0.05 (i.e. sex, age, BMI, exercise habits, coffee and tea consumption, education level, residential urbanicity, family history and sleeping profiles (including SJL and sleep quality)) were controlled for in a subsequent multivariate analysis. Definitions of covariates are listed in Supplementary Table 1.

### Influence of tobacco and alcohol use on anxiety

Those participants who were non-users of both tobacco and alcohol formed the reference group, which was compared with those who were tobacco-only users, alcohol-only users or users of both tobacco and alcohol. All variables with significant results (*P* < 0.05) in our univariate analysis were selected as covariates for our logistic regression analysis. Tobacco-only users, alcohol-only users and users of both tobacco and alcohol were compared with the reference group (non-users of both tobacco and alcohol), and were revealed as being more prone to developing anxiety (adjusted odds ratio (aOR) with 95% CI: tobacco-only users, 1.30 [1.08–1.56]; aOR with 95% CI: alcohol-only users, 1.51 [1.09–2.10]; aOR with 95% CI: users of both tobacco and alcohol, 1.77 [1.36–2.29]). Several covariates (i.e. age, sex, exercise habits, tea consumption and sleep quality) were significantly correlated with a reduced risk of anxiety (aOR [95% CI]: age ≥55 years, 0.60 [0.52–0.69]; tea consumption, 0.84 [0.72–0.98]; regular exercise, 0.69 [0.60–0.79]; and good^+^ sleep quality, 0.28 [0.25–0.32]) By contrast, female sex was significantly correlated with an increased risk of anxiety (aOR [95% CI]: 1.91 [1.60–2.27]). These results are listed in Table 2.

**Table 2:** Effect of alcohol drinking and smoking behaviours on risk of anxiety

Independent variables	Adjusted OR (95% CI)	*P*-value
Alcohol and tobacco use (versus absence of alcohol and tobacco use)		
Tobacco-only users	1.30 (1.08–1.56)	0.006**
Alcohol-only users	1.51 (1.09–2.10)	0.014*
Users of both alcohol and tobacco	1.77 (1.36–2.29)	<0.001***
Age (≥55 *v*. <55 years)	0.60 (0.52–0.69)	<0.001***
Female (versus male)	1.91 (1.60–2.27)	<0.001***
Residential urbanicity (rural *v*. non-rural)	1.16 (0.89–1.52)	0.266
Education level (versus high school, elementary school or lower level of education)		
College or graduate school	1.08 (0.95–1.23)	0.228
Baseline comorbidity		
BMI ≥25 (*v*. BMI <25)	0.91 (0.80–1.04)	0.158
Behavioural factors		
Regular exercise (versus absence of regular exercise)	0.69 (0.60–0.79)	<0.001***
Coffee consumption (versus absence of coffee consumption)	0.90 (0.80–1.02)	0.106
Tea consumption (versus absence of tea consumption)	0.84 (0.72–0.98)	0.028*
Social jet lag between weekdays and weekends		
Difference in sleep hours (*v*. <1 h)	1.08 (0.94–1.25)	0.257
Sleep quality (good^+^ versus bad^+^)	0.28 (0.25–0.32)	<0.001***
Presence of mental illness (versus absence of mental illness)	1.14 (0.84–1.53)	0.407

BMI, body mass index; CI, confidence interval; OR, odds ratio.

*P < 0.05, **P < 0.01, ***P < 0.001.

### Sex-stratified difference in influence of tobacco and alcohol use on anxiety

According to a sex-stratified analysis among women, the use of tobacco only (aOR [95% CI], 1.33 [1.04–1.70]) and the use of both tobacco and alcohol (2.12 [1.31–3.43]) were associated with higher anxiety risk relative to their non-use. Among men, only the use of both tobacco and alcohol (1.67 [1.19–2.33]) was associated with a higher anxiety risk relative to their non-use. For both male and female participants, several covariates (e.g. age ≥55 years, regular exercise and sleep quality) were significantly correlated with a reduced risk of anxiety (aOR [95% CI]: male participants aged ≥55 years, 0.50 [0.38–0.6]; female participants aged ≥55 years, 0.61 [0.49–0.76]; male participants who exercised regularly, 0.69 [0.53–0.91]; female participants who exercised regularly, 0.69 [0.59–0.81]; male participants with good^+^ sleep quality, 0.29 [0.23–0.37]; and female participants with good^+^ sleep quality, 0.28 [0.24–0.32]). Those male participants who drank tea had a significantly lower risk of anxiety (aOR [95% CI]: 0.74 [0.56–0.97]), whereas those with a family history of mental illness had a significantly higher risk of anxiety (aOR [95% CI]: 2.05 [1.12–3.75]). These results are listed in Table 3.

**Table 3:** Sex-stratified effects of alcohol drinking and smoking behaviours on risk of anxiety

Independent variables	Men Adjusted OR (95% CI)	*P*-value	Women Adjusted OR (95% CI)	*P*-value
Alcohol and tobacco use (versus absence of alcohol and tobacco use)				
Tobacco-only users	1.28 (0.95–1.72)	0.109	1.33 (1.04–1.70)	0.024*
Alcohol-only users	1.64 (0.96–2.81)	0.071	1.42 (0.93–2.16)	0.101
Users of both alcohol and tobacco	1.67 (1.19–2.33)	0.003**	2.12 (1.31–3.43)	0.002**
Age (*v*. <55 years)	0.50 (0.38–0.65)	<0.001***	0.61 (0.49–0.76)	<0.001***
Menopausal status (versus premenopausal)				
Postmenopausal			1.08 (0.87–1.35)	0.471
Residential urbanicity (rural versus non-rural)	1.10 (0.67–1.79)	0.708	1.18 (0.86–1.63)	0.305
Educational level (versus high school, elementary school or lower level of education)				
College or graduate school	1.03 (0.79–1.34)	0.836	1.11 (0.96–1.29)	0.166
Baseline comorbidity				
BMI ≥25 (*v*. BMI <25)	0.92 (0.72–1.18)	0.523	0.90 (0.77–1.05)	0.186
Behavioural factors				
Regular exercise (versus no regular exercise)	0.69 (0.53–0.91)	0.007**	0.69 (0.59–0.81)	<0.001***
Coffee-drinking habits (versus no coffee consumption)	1.01 (0.79–1.29)	0.926	0.87 (0.75–1.00)	0.051
Tea-drinking habits (versus no tea consumption)	0.74 (0.56–0.97)	0.031*	0.90 (0.75–1.09)	0.286
Social jet lag between weekdays and weekends				
Difference in sleep hours (*v*. <1 h)	0.94 (0.71–1.25)	0.693	1.14 (0.97–1.34)	0.109
Sleep quality (good^+^ versus bad^+^)	0.29 (0.23–0.37)	<0.001***	0.28 (0.24–0.32)	<0.001***
Presence of mental illness (versus absence of mental illness)	2.05 (1.12–3.75)	0.020*	0.98 (0.69–1.38)	0.896

BMI, body mass index; CI, confidence interval; OR, odds ratio.

*P < 0.05, **P < 0.01, ***P < 0.001.

### Influence of tobacco and alcohol use on anxiety in men across age groups

We compared the results of male participants aged <55 and ≥55 years. In both age groups, participants who were users of both tobacco and alcohol were more prone to having anxiety than were those who were users of neither product (aOR [95% CI]: male participants aged <55 years with tobacco and alcohol use, 1.64 [1.08–2.49]; and male participants aged ≥55 years with tobacco and alcohol use, 1.84 [1.03–3.27]). Among male participants aged ≥55 years, those who were tobacco-only users were more prone to having anxiety (aOR [95% CI]: 1.84 [1.12–3.04]); this significant difference was not identified in male participants aged <55 years. In both age groups, male participants with good^+^ sleep quality were less prone to having anxiety than were those without (aOR [95% CI]: male participants aged <55 years with good^+^ sleep quality, 0.24 [0.17–0.33]; and male participants aged ≥55 years with good^+^ sleep quality, 0.40 [0.26–0.60]). Notably, male participants aged <55 years were more prone to having anxiety if they lived in a rural area. By contrast, male participants aged ≥55 years were less prone to having anxiety if they lived in a rural area (aOR [95% CI]: male participants aged <55 years who lived in a rural area, 2.76 [1.12–6.81]; and male participants aged ≥55 years who lived in a rural area, 0.49 [0.27–0.90]). Those male participants aged <55 years and who had a family history of mental illness were significantly more prone to having anxiety than were those without such a history (aOR [95% CI]: male participants aged <55 years with a family history of mental illness, 2.19 [1.02–4.67]). This significant difference was not identified in male participants aged ≥55 years. Those male participants aged ≥55 years who exercised regularly or consumed tea were significantly less prone to having anxiety than were those who did not engage in these activities (aOR [95% CI]: male participants aged ≥55 years who exercised regularly, 0.67 [0.45–1.00]; and male participants aged ≥55 years who consumed tea, 0.51 [0.32–0.82]). These significant differences were not identified in male participants aged <55 years. These results are listed in Table 4.

**Table 4:** Age-stratified effects of alcohol drinking and smoking behaviours on risk of anxiety in men (<55 *v*. ≥55 years)

Independent variables	Men aged <55 years Adjusted OR (95% CI)	*P*-value	Men aged ≥55 years Adjusted OR (95% CI)	*P*-value
Alcohol and tobacco use (versus no alcohol or tobacco use)				
Tobacco-only users	1.02 (0.70–1.50)	0.904	1.84 (1.12–3.04)	0.016*
Alcohol-only users	1.55 (0.79–3.03)	0.202	1.89 (0.76–4.70)	0.170
Users of both alcohol and tobacco	1.64 (1.08–2.49)	0.021*	1.84 (1.03–3.27)	0.038*
Residential urbanicity (rural versus non-rural)	2.76 (1.12–6.81)	0.028*	0.49 (0.27–0.90)	0.021*
Educational level (versus high school, elementary school or lower level of education)				
College or graduate school	1.16 (0.81–1.68)	0.416	0.88 (0.59–1.32)	0.540
Baseline comorbidity				
BMI ≥ 25 (*v.* BMI < 25)	0.85 (0.62–1.17)	0.317	1.03 (0.70–1.53)	0.880
Behavioural factors				
Regular exercise (versus no regular exercise)	0.72 (0.50–1.04)	0.084	0.67 (0.45–1.00)	0.049*
Coffee-drinking habits (versus no coffee consumption)	1.15 (0.84–1.57)	0.396	0.83 (0.55–1.25)	0.377
Tea-drinking habits (versus no tea consumption)	0.89 (0.63–1.27)	0.527	0.51 (0.32–0.82)	0.006**
Social jet lag between weekdays and weekends				
Difference in sleep hours (*v*. <1 h)	0.94 (0.68–1.30)	0.717	0.86 (0.46–1.63)	0.651
Sleep quality (good^+^ versus bad^+^)	0.24 (0.17–0.33)	<0.001***	0.40 (0.26–0.60)	<0.001***
Presence of mental illness (versus absence of mental illness)	2.19 (1.02–4.67)	0.043*	1.78 (0.64–4.94)	0.271

BMI, body mass index; CI, confidence interval; OR, odds ratio.

*P<0.05, **P<0.01, ***P<0.001.

### Influence of tobacco and alcohol use on anxiety in women across age groups

Among female participants aged <55 years, anxiety risk was not significantly associated with the use of tobacco or alcohol or both. Among female participants aged <55 years, only the use of both tobacco and alcohol was associated with higher anxiety risk (aOR [95% CI]: 2.18 [1.24–3.85]). In both age groups, those female participants with regular exercise or good^+^ sleep quality were less prone to having anxiety relative to those without regular exercise or without good^+^ sleep quality (aOR [95% CI]: female participants aged <55 years with regular exercise, 0.77 [0.61–0.96]; female participants aged ≥55 years with regular exercise, 0.63 [0.50–0.78]; aOR [95% CI]: female participants aged <55 years with good^+^ sleep quality, 0.29 [0.24–0.34]; and female participants aged ≥55 years with good^+^ sleep quality, 0.27 [0.22–0.34]). These results are listed in Table 5.

**Table 5:** Age-stratified effects of alcohol drinking and smoking behaviours on risk of anxiety in women (<55 *v*. ≥55 years)

Independent variables	Women aged <55 years Adjusted OR (95% CI)	*P*-value	Women aged ≥55 years Adjusted OR (95% CI)	*P*-value
Alcohol and tobacco use (versus no alcohol and tobacco use)				
Tobacco-only users	1.29 (0.97–1.72)	0.077	1.45 (0.89-2.39)	0.139
Alcohol-only users	1.26 (0.71–2.26)	0.430	1.67 (0.91–3.06)	0.095
Users of both alcohol and tobacco	2.18 (1.24–3.85)	0.007**	1.88 (0.74–4.78)	0.184
Menopausal status (versus premenopausal)				
Postmenopausal	1.12 (0.90–1.41)	0.312	0.67 (0.31–1.47)	0.321
Residential urbanicity (rural versus non-rural)	1.37 (0.90–2.08)	0.141	0.91 (0.55–1.51)	0.713
Educational level (versus high school, elementary school or lower level of education)				
College or graduate school	1.21 (0.99–1.48)	0.066	1.00 (0.79–1.27)	0.991
Baseline comorbidity				
BMI ≥ 25 (*v*. BMI < 25)	1.00 (0.82–1.23)	0.980	0.78 (0.61–1.00)	0.046*
Behavioural factors				
Regular exercise (versus no regular exercise)	0.77 (0.61–0.96)	0.020*	0.63 (0.50–0.78)	<0.001***
Coffee-drinking habits (versus no coffee consumption)	0.91 (0.76–1.10)	0.327	0.81 (0.64–1.02)	0.071
Tea-drinking habits (versus no tea consumption)	0.98 (0.77–1.23)	0.839	0.79 (0.57–1.08)	0.141
Social jet lag between weekdays and weekends				
Difference in sleep hours (*v*. <1 h)	1.13 (0.94–1.37)	0.192	1.14 (0.84–1.55)	0.402
Sleep quality (good^+^ versus bad^+^)	0.29 (0.24–0.34)	<0.001***	0.27 (0.22–0.34)	<0.001***
Presence of mental illness (versus absence of mental illness)	0.77 (0.47–1.27)	0.306	1.25 (0.77–2.01)	0.363

BMI, body mass index; CI, confidence interval; OR, odds ratio.

*P < 0.05, **P < 0.01, ***P < 0.001.

## Discussion

We investigated the influence of tobacco and alcohol use on anxiety by analysing data retrieved from the TWB, thereby addressing several literature gaps. First, we discovered that those participants who were tobacco-only users, alcohol-only users or users of both tobacco and alcohol were more prone to having anxiety than was the reference group (those participants who were non-users of both tobacco and alcohol). Several studies have also discussed this topic. One of the most convincing hypotheses is based on the stress-coping model, which posits that individuals who experience more stress are more prone to using tobacco, alcohol or other substances as emotional regulators.^25^

Second, our sex-stratified results indicated that women were significantly more prone to having anxiety (Table 2) and that those men who were tobacco-only users, alcohol-only users or users of both tobacco and alcohol were significantly more prone to having anxiety than men who were not users. By contrast, among female participants, only those who were users of tobacco and alcohol were significantly more prone to having anxiety relative to the reference group (Table 3). Although female participants were significantly more prone to having anxiety relative to male participants, the effects of tobacco and alcohol use on anxiety were less frequently observed in the former.^26^ This finding may also have been due to the greater susceptibility of women to anxiety (Table 2). By contrast, male participants were more prone than female participants to using tobacco and alcohol when they experienced anxiety (Table 3). These topics are highly relevant and warrant further investigation, because the prevalence of tobacco and alcohol use among women has been increasing in Taiwan in recent years.^27,28^

Third, we compared participants on the basis of age (<55 *v*. ≥55 years). The results indicate that older age is a protective factor against anxiety (Tables 2 and 3): that is, older participants were less prone to having anxiety. This finding is consistent with those of another study that investigated a sample of adults in the USA, and reported that the prevalence of anxiety disorders was 34.2% among individuals aged 45–59 years but significantly lower (at 17.8%) among individuals aged >60 years.^29^ Although the age grouping of that study differs from that of ours, the differences with respect to age and the tendency to develop anxiety disorders are similar.

In addition to tobacco and alcohol use, other covariates were used to adjust our statistical analysis. Covariates such as exercise habits and sleep quality were examined as potential protective factors, and our results are consistent with those of other studies. Specifically, our results suggest that regular physical exercise is a protective factor against anxiety and is associated with a lower prevalence of anxiety.^30^ They also suggest that regular physical exercise reduces or even treats symptoms of anxiety.^30,31^ The mechanism underlying the relationship between anxiety and physical activity remains unclear. The positive and significant effect of exercise on anxiety in older adults was also reported in other studies.^32,33^ Several studies have reported that poor sleep quality increased the odds ratio of having anxiety and various other mental disorders.^15,34,35^ In addition to alcohol and tobacco use, various studies have also identified other risk and protective factors that can modify or influence the development of depression and anxiety in adolescents.^36,37^ However, the evidence supporting the existence of modifiable risk factors for anxiety in adults is limited. The results of our covariate analysis suggest that interventions focused on regular exercise and sleep habits should be developed and implemented for people with anxiety.

In the present study, the risk of anxiety was considerably higher among users of tobacco or alcohol than among non-users. Although women exhibited a higher risk of anxiety than did men, the influence of alcohol and tobacco use on anxiety was greater in men than in women. Our analysis indicates that considerably fewer women use tobacco and alcohol than do men (Table 1). However, the associations of anxiety with tobacco and alcohol use in women may change because the prevalence of women using tobacco and alcohol in Taiwan has increased markedly.

### Limitations

The present study adopted a cross-sectional observational design. Because physical illness records, including those related to medication use, were not documented in the database, their influence on anxiety could not be measured in our study. We analysed responses to the first two questions in PHQ-4 from the included participants to determine whether they had anxiety. Because the first two questions comprise a series of questionnaires and a scoring system designed to measure the risk of anxiety, enquiring into the presence of anxiety symptoms only, results from clinical diagnoses were not examined in the present study. Because the first two questions in PHQ-4 comprise a questionnaire and scoring system designed to assess the risk of anxiety, evaluating the presence of anxiety symptoms only, clinical diagnosis results were not examined in this study. To confirm the associations of anxiety disorders with tobacco and alcohol use, the definition of anxiety should be reconsidered. Additionally, those individuals enrolled in the TWB completed baseline questionnaires that recorded alcohol drinking and tobacco smoking only as categorical outcomes. Because we recruited individuals with baseline conditions in our study, we could not assess any potential dose–response effect or determine the effect of the number of years of alcohol or tobacco consumption.

## Supporting information

Wen et al. supplementary material Wen et al. supplementary material

## Data Availability

The data supporting the findings of this study are available upon reasonable request from the Taiwan Biobank (TWB). The TWB restricts public access to its data.

## Supplementary material

The supplementary material is available online at https://doi.org/10.1192/bjo.2025.24
