# Supplementary material for: Influence of tobacco smoking and alcohol drinking on anxiety in sample of 30 836 individuals in Taiwan Biobank
Source: BJPsych Open. 2025 Apr 1;11(3):e78. doi: 10.1192/bjo.2025.24 (PMC12052577; doi:10.1192/bjo.2025.24)
Supplement: Wen et al. supplementary material [file S2056472425000249sup001.docx]

Table 1. The definition of study variables.

| **Variables** | **Definition** |
| --- | --- |
| Residential urbanicity | Residential urbanicity was classified into rural (clusters 5-7) and non-rural (clusters 1-4) according to the seven urbanization stratification clusters (1, most urbanized; 7, least urbanized) as defined by Liu et al.^a^ |
| Education level (vs. high school, elementary school, or lower level of education) | Education was classified into three levels (1) college or graduate school, 2) high school, 3) elementary school or lower level of education) |
| Regular exercise | Regular exercise was defined as exercising at least 3 times a week exceeding 30 minutes each time. |
| Coffee consumption | Habitual coffee drinkers. |
| Tea consumption | Habitual tea drinkers. |
| Social jet lag between weekdays and weekends | Social jet lag is defined as the differences in sleep hours of two or more hours between weekdays and weekends |
| Sleep quality | Sleep quality was measured using a 5-point Likert scale item from 1 (very bad quality), 2 (bad quality), 3 (normal quality), 4 (good quality) to 5 (very good quality). We dichotomized the sleep quality into good sleeping quality (3-5) and bad sleeping quality (1 and 2). |
| Family history of mental illness | Family history of mental illness refers second-degree relatives with depression, manic depression, postpartum depression, obsessive compulsive disorder, alcoholism drug abuse or schizophrenia. |
| Menopausal status, (vs. premenopausal) | Self-reported menopausal status by women. |

^a^Liu C-Y, Hung Y-T, Chuang Y-L, Chen Y-J, Weng W-S, Liu J-S, et al. Incorporating Development Stratification of Taiwan Townships into Sampling Design of Large Scale Health Interview Survey (*in Chinese*)*.* Journal of Health Management 2006;4:1-22.
